# *Burkholderia cepacia* Infections at Sites Other than the Respiratory Tract: A Large Case Series from a Tertiary Referral Hospital in Lebanon

**DOI:** 10.1007/s44197-022-00048-2

**Published:** 2022-06-30

**Authors:** Rola Kwayess, Housam Eddine Al Hariri, Joya-Rita Hindy, Nada Youssef, Sara F. Haddad, Souha S. Kanj

**Affiliations:** 1 Department of Internal Medicine, American University of Beirut Medical Center, Beirut, Lebanon; 2 Division of Infectious Diseases, American University of Beirut Medical Center, Riad El Solh, PO Box 11-0236, Beirut, 1107 2020 Lebanon

**Keywords:** *Burkholderia cepacia*, Osteomyelitis, Bacteremia, Skin and soft tissue infections

## Abstract

**Objectives:**

The *Burkholderia cepacia* complex (Bcc), which was originally thought to be a single species, represents a group of 24 distinct species that are often resistant to multiple antibiotics, and usually known to cause life-threatening pulmonary infections in cystic fibrosis patients. Herein we describe a series of non-respiratory Bcc infections, the risk factors and epidemiologic factors, in addition to the clinical course.

**Patients and methods:**

This is a retrospective chart review of 44 patients with documented *B. cepacia* infections isolated from sites other than the respiratory tract admitted between June 2005 and February 2020 to the American University of Beirut Medical Center (AUBMC), a tertiary referral hospital for Lebanon and the Middle East region. The epidemiological background of these patients, their underlying risk factors, the used antibiotic regimens, and the sensitivities of the *B. cepacia* specimens were collected.

**Results:**

The majority of the Bcc infections (26/44, 59.1%) were hospital-acquired infections. The most common nationality of the patients was Iraqi (18/44, 40.9%), and the most common site of infection was bacteremia (17/44, 38.6%), followed by skin and soft tissues infections (16/44, 36.4%) and vertebral osteomyelitis (8/44, 18.2%). Most of the isolated *B. cepacia* were susceptible to ceftazidime, carbapenems, followed by TMP-SMX. Patients responded well to therapy with good overall outcome.

**Conclusions:**

Bcc can cause infections outside the respiratory tract, mostly as hospital-acquired infections and in immunocompromised patients. Most patients were referred from countries inflicted by wars raising the possibility of a potential role of conflicts which need to be investigated in future studies. Directed therapy according to susceptibility results proved effective in most patients.

## Introduction

The *Burkholderia cepacia* complex (Bcc) represents a group of 24 distinct species [1]. These Gram-negative rods are motile, aerobic, glucose non-fermenting organisms, and often resistant to multiple antibiotics. *B. cepacia* is a common respiratory pathogen in patients with chronic lung disease such as cystic fibrosis [2] but rarely causes community-acquired infections outside the respiratory tract. Organisms of the Bcc are found in natural habitats such as soil and water. Because they can survive in the presence of disinfectants, they act as potential nosocomial infections. Multiple studies suggest that patients with debilitating underlying diseases, those with an indwelling device or those with prolonged intensive care unit stay, are at increased risk of Bcc nosocomial infections [3–5].

Bcc infections are intrinsically resistant to many antimicrobial agents thus limiting the therapeutic options. Although Bcc has been rarely reported as the causative organism of vertebral osteomyelitis, its overall incidence in this location has steadily increased during the past years [6–8]. Some antibiotics such as ceftazidime, carbapenem, trimethoprim-sulfamethoxazole (TMP-SMX), and fluoroquinolones have shown in vitro activities against this bacterium, and some combinations of antibiotics have shown synergy [5].

The rarity of published Bcc infections outside the respiratory tract in the literature highlights the importance of this study where we report the unusual increased number of infections outside the respiratory tract over the past few years at our medical center particularly among patients referred from Syria and Iraq, countries inflicted by wars.

## Methods

### Study Design and Setting

This study is a retrospective chart review of 44 patients who presented to the American University of Beirut Medical Center (AUBMC), the largest tertiary care center in Lebanon, with documented *B. cepacia* complex infection at sites other than the respiratory tract reviewed between June 2005 and February 2020. The epidemiological background of these patients, their underlying risk factors and comorbidities, the antibiotic regimens used, the duration of their treatment as well as the sensitivities of the Bcc isolates were reviewed (Table 1).

**Table 1:** Characteristics of patients with *B. cepa*cia infection

Type of Infection	Age	Sex	Nationality	Hospital Admission	ICU Admission	Infection Acquisition	Treatment and Duration*	Disposition
Bacteremia	73	M	L	Yes	No	HAI	Carbapenem for 8 days	Discharged
	59	M	L	Yes	Yes	HAI	Carbapenem for 10 days	
	59	M	I	Yes	No	HAI	Carbapenem for 10 days	
	34	M	S	Yes	Yes	HAI	Carbapenem for 14 days	
	77	M	L	Yes	No	HAI	Quinolone for 15 days	
	53	F	L	Yes	Yes	HAI	Ceftazidime for 10 days	
	89	M	L	Yes	No	CAI	Ceftazidime for 14 days	
	69	F	L	Yes	No	HAI	TMP-SMX and Quinolone for 18 days	
	40	M	L	Yes	No	HAI	NA	
	70	F	S	Yes	No	HAI	Ceftazidime and Tetracycline for 10 days	
	7	M	S	Yes	Yes	HAI	TMP-SMX for 12 days	
	54	M	I	Yes	Yes	HAI	Ceftazidime and Aminoglycoside for 15 days	
	43	M	I	Yes	No	HAI	Aminoglycoside and TMP-SMX for 14 days	Discharged
	69	M	I	Yes	Yes	CAI	Piperacillin-tazobactam and Vancomycin for 15 days	
	63	F	S	Yes	Yes	HAI	Ceftazidime and Quinolone for 37 days	Deceased
Bacteremia + Osteomyelitis	33	F	I	Yes	No	HAI	Tetracyclineand Rifampin for 21 days	Discharged
	43	M	I	No	No	CAI	NA	NA
Osteomyelitis	58	F	I	Yes	No	HAI	Ceftazidime and TMP-SMX for 42 days	Discharged
	28	F	I	Yes	No	CAI	Ceftazidime for 14 days	
	99	M	NA	Yes	No	HAI	Tigecycline and Quinolone for 10 days	
	56	F	S	No	No	CAI	Quinolone	NA
	36	M	I	No	No	CAI	TMP-SMX for 210 days	
	30	F	I	No	Yes	NA	Quinolone	
	40	M	I	No	Yes	CAI	Ceftazidime, TMP-SMX, and Quinolone for 28 days	
	36	M	S	No	Yes	HAI	Ceftazidime, Tetracycline, and Quinolone for 60 days	
Skin/soft tissue	25	M	L	No	No	CAI	Tigecycline, TMP-SMX, and Quinolone for 7 days	NA
	22	M	L	Yes	No	HAI	Ceftazidime, Tigecycline, and Quinolone for 17 days	Discharged
	71	M	L	Yes	No	CAI	TMP-SMX for 14 days	
	31	M	L	No	No	NA	NA	
	13	M	I	Yes	Yes	HAI	Tigecycline for 20 days	
Abscess	56	M	L	Yes	No	HAI	Carbapenem	Discharged
	58	M	L	Yes	No	HAI	Carbapenem for 12 days	
	26	F	I	Yes	Yes	CAI	Ceftazidime for 14 days	
	56	M	I	Yes	No	HAI	Carbapenem for 18 days	
Wound	46	M	L	Yes	No	HAI	Quinolone and Vancomycin	Discharged
	56	M	S	No	No	NA	NA	
	65	M	I	Yes	No	CAI	Carbapenem	
Catheter	51	F	I	No	No	CAI	NA	NA
	50	F	S	Yes	No	CAI	Ceftazidime	Discharged
Other	53	M	S	Yes	No	CAI	NA	Discharged
	56	M	L	Yes	No	HAI	Ceftazidime	
	47	M	I	Yes	No	CAI	TMP-SMX	
	49	M	I	Yes	No	HAI	Quinolone	
	66	M	I	Yes	No	NA	Minocycline	NA

*M* male, *F* female, *L* lebanese, *S* Syrian, *I* Iraqi, *HAI* hospital acquired infection, *CAI* community acquired infection

^*^The standard dose for treatment of gram-negative bacteria was used, adjusted to creatinine clearance

### Microbiological Data

*B. cepacia* was identified as per standardized laboratory methods, cultured on MacConkey agar. The antibiotics tested for susceptibility were chosen by the microbiology laboratory at AUBMC as per the yearly updated recommendations of the Clinical Laboratory and Standards Institute (CLSI) [9] and included minocycline, TMP-SMX, meropenem, ceftazidime, with standardized disc diffusion (DD) testing methods. As for ciprofloxacin, aminoglycoside, tigecycline, and piperacillin-tazobactam, they were tested upon physician’s request.

### Clinical Data

The patients’ demographics, comorbidities, smoking status, potential risk factors such as the presence of indwelling catheters at the time of the positive cultures, prior procedures, the use of steroids or any other therapy causing immunosuppression within 30 days prior to the positive cultures were recorded from the electronic health records (EHR).

The type and source of the Bcc infection were identified by reviewing the dates of positive cultures. The antibiotics administered prior to the culture results for other infections as well as the antibiotics administered after the cultures grew Bcc were collected.

### Criteria for Bcc Classification

Hospital acquired infections were defined as infections with an onset of > 48 h since current admission or < 90 days since last hospitalization, while infections occurring as outpatient or < 48 h since current admission were designated as community acquired infections.

Patients with *B. cepacia* bacteremia as a complication of respiratory infections were excluded.

Patients who had received chemotherapy, immunotherapy, biologic therapy, radiation therapy or steroids within the 30 days prior to their current admission were considered immunosuppressed.

The Pitt bacteremia score [10], the Charlson index score [11], and the SOFA score [12] were calculated for patients with bacteremia.

### Statistical Analysis

The Statistical Package for Social Sciences (SPSS), version 27, was used to perform data entry, management, and analyses. The descriptive of the continuous and categorical variables as means/standard deviations and frequencies/percentages were presented, respectively.

## Results

### Patients’ Characteristics

The mean age of these 44 patients was 50 + / 19 years at the time of their Bcc infection and 31 of them (31/44, 70.5%) were male. The most common nationality was Iraqi (18/44, 40.9%), followed by Lebanese (15/44, 34.1%), and Syrian (9/44, 20.5%) nationalities. 65.9% (29/44) of our patients were non-Lebanese residents. Throughout the dates of the patients’ admissions the percentage of Iraqi and Syrian patients at AUBMC did not exceed 2–3%. Thirty-eight patients (38/44, 86.4%), had a history of malignancy (19/44, 50%) or a history of surgical procedure on the infection site (19/44, 50%). Eight patients (8/44, 18.2%) had atherosclerotic cardiovascular disease, 14 (14/44, 31.8%) were smokers and 7 (7/44, 15.9%) were diabetic. Sixteen patients (16/44, 36.4%) had received antibiotics within the 30 days prior to the isolation of Bcc for other types of infections. The two most commonly used antibiotics were ceftazidime and carbapenems followed by quinolones. Nine patients (9/44, 20.5%) received therapies other than antibiotics within the 30 days prior to their infection, specifically chemotherapy and steroids (Table 2).

**Table 2:** Risk factors of patients with *B. cepa*cia infections

Patients’ characteristics	
Age	50.34 ± 19.048
Gender	
Males	31 (70.5%)
Females	13 (29.5%)
Nationality	
Iraqi	18 (40.9%)
Lebanese	15 (34.1%)
Syrian	9 (20.5%)
Others	1 (4.5%)
Past medical history	
Diabetes Mellitus	7 (15.9%)
Smoking history	26 (59.1%)
Atherosclerotic cardiovascular disease	8 (18.2%)
Cerebral vascular accident	3 (6.8%)
Chronic kidney disease	6 (13.6%)
Chronic liver disease	2 (4.5%)
Malignancy	19 (43.2%)
Prior surgical history on affected site	19 (43.2%)
History of trauma	4 (9.1%)

### Hospital Admissions’ Characteristics

Thirty-three patients (33/44, 75%) were admitted to the hospital at the time they had their positive Bcc culture, with a median length of hospital stay of 8 (5–16.5) days. At the time of the positive culture, 20 of these admitted patients (20/44, 45.5%) had an indwelling catheter and 12 (12/44, 27.3%) were in the ICU, with a median length of ICU stay of 3.5 (2–10.5) days. Thirty-two patients (32/44, 96.9%) were discharged from the hospital, whereas one patient succumbed to his infection.

### *Burkholderia cepacia* Complex Infections’ Characteristics

Almost all the Bcc infections (43/44, 97.7%) were recorded in these patients for the first time, where 26 of them (26/44, 59.1%) were hospital-acquired infections. Multiple sites of Bcc infections were noted, with the most common being central line associated bacteremia (17/44, 38.6%), followed by skin and soft tissue infections (SSTIs) (including ulcers, wounds, and burns) as well as deep seated cysts and abscesses (16/44, 36.4%) and vertebral osteomyelitis (8/44, 18.2%). Four other infection sites were noted and included, ascitic fluid, vitreous fluid, gastric band port, a scalp lesion and biliary fluid, respectively. In the patients with bacteremia, the average Pitt Bacteremia score, SOFA score, and Charlson index score were respectively 1.36 (± 2.678), 3.27 (± 3.349), and 4.40 (± 3.641). Among the eight vertebral osteomyelitis cases, four were located in the lumbar region, three in the cervical region and one at multiple vertebral levels; five of these cases were native vertebral osteomyelitis.

### *Burkholderia cepacia* Specimens’ Susceptibilities

The percentages of susceptibility to ceftazidime, tetracycline, trimethoprim-sulfamethoxazole and carbapenems were 84.1% (37/44), 54.5% (24/44), 63.2% (28/44) and 65.9% (29/44), respectively. Only eight isolates were tested for susceptibility to quinolones, and out of these; three were resistant. In addition, two out of the four specimens that were tested for susceptibility to piperacillin-tazobactam were resistant.

### Patients’ Management

Antibiotic regimens were administered at our center depending on the clinician’s expertise, the patient’s comorbidities, and his clinical presentation. Once the cultures grew *B. cepacia*, the patients received directed therapy, with regimens including one or more of the following antibiotics: ceftazidime, quinolones, carbapenems, TMP-SMX, for an average duration of 23 days. Out of the 17 patients with bacteremia, 10 patients (10/17, 58.8%) were treated with a single antibiotic, 5 patients (5/17, 29.4%) received a combination therapy with 2 antibiotics, and only one patient (1/17, 5.9%) was treated with 3 antibiotics. Out of the 16 patients with SSTIs (including ulcers, wounds, and burns) as well as deep seated cysts and abscesses, 10 patients (10/16, 62.5%) received a single antibiotic, and 2 patients (2/16, 12.5%) were treated with a combination of two antibiotics. Out of the eight patients with osteomyelitis, four of them (4/8, 50%) were treated with a single antibiotic, three patients (3/8, 37.5%) received a combination therapy with two antibiotics, and one patient (1/8, 12.5%) was treated with three antibiotics. Among these patients, only two presented with neurological symptoms that required vertebral decompression by partial corpectomy. After the initiation of directed antibiotics, none of the eight patients with vertebral osteomyelitis required surgical debridement.

## Discussion

Infections with Bcc outside the respiratory tract are uncommon. Our reported cases were mostly hospital acquired and had bacteremia, infected cysts and osteomyelitis. Of particular concern are the cases of osteomyelitis requiring prolonged antibiotic therapy.

The Infectious Diseases Society of America (IDSA) has recently published guidelines for the management of resistant Gram negative infections [13]. Unfortunately, no guidance on the proper management of *B. cepacia* was included. We have historically relied on the management of such infections based on clinical experience and published case reports. Few cases of Bcc osteomyelitis have been reported in the literature to date. Weinstein et al*.* reported a case of a 49-year-old female patient who presented 3 weeks after a rhinoplasty procedure, with *B. cepacia* osteomyelitis. Susceptibility testing revealed that the specimen was susceptible to ceftazidime, imipenem-cilastatin, levofloxacin, meropenem and piperacillin. The patient was treated with meropenem for 6 weeks with gradual resolution of her neck pain [14]. Another case of a 71-year-old previously healthy female farmer who presented with back pain and was found to have osteomyelitis at T5 level was reported by Hsieh et al*.* Initially, cefazolin and gentamicin were prescribed. She then developed neurologic signs necessitating transthoracic corpectomies. Intraoperative tissue culture confirmed *B. cepacia* infection which was susceptible to ceftazidime, meropenem, TMP-SMX, and levofloxacin. The source of the patient’s *B. cepacia* infection was thought to be her occupational environment. Her antibiotic therapy was changed to levofloxacin afterwards; however, she did not survive eventually due to septic shock [15]. In addition, Al Attia et al*.* reported a case of a 52-year-old presenting cranial vault osteomyelitis and multiple ring-enhancing frontal lobe brain abscesses. Cultured pus grew *B. cepacia* and he was treated with a combination of ceftazidime and piperacillin-tazobactam with marked improvement of his intracerebral and scalp lesions 3 weeks later [7]. Moreover, Lee et al*.* reported a case of an 80-year-old female patient who was found to have osteomyelitis of the sternum after a traumatic injury. Blood and pus cultures grew *B. cepacia* sensitive to ceftazidime, meropenem, TMP-SMX, and tigecycline. She was initially treated with ceftazidime followed by TMP-SMX and oral moxifloxacin for 3 months and improved initially. However, after having recurrence of the pus discharge, she received tigecycline, plus TMP-SMX with no documented recurrence thereafter [5].

Most of the reported patients in this series were Iraqi and Syrian despite the fact that the percentage of non-Lebanese patients at our hospital did not exceed 2–3% at any time during the study period. This raises the concern that countries at war might be at increased risk for such infections. Although there are no published data suggesting this to date, this needs to be further studied as we are currently witnessing more wars and conflicts in various parts of the world. Some other organisms such as *Acinetobacter* sp. had been previously associated with wars [16–20]. Recent data suggest that such organisms might be associated with heavy metals contamination [21]. Whether the same occurs with *B. cepacia* needs to be investigated. Most of the patients had risk factors such as malignancies, diabetes mellitus, recent steroids use, recent antibiotic use, including those not known to be active against Bcc, and recent surgical intervention at the site of the *B. cepacia* infection. The latter has most likely been related to contaminated solutions or other materials used intraoperatively as previously reported [8, 22]. Other reported sources of hospital-acquired Bcc infections include intravenous flush solutions, contaminated ultrasound gel, albuterol and nasal spray [6–8]. However, this was unlikely in our cases as we could not identify a cluster of cases.

There have been some variations in the sensitivities based on the type of susceptibility testing used. Though EUCAST does not recommend to test for Bcc susceptibility routinely in diagnostic microbiology laboratories because of poor reproducibility and correlation in all methods used, disk diffusion is recommended by CLSI, [23] and is the method adapted at AUBMC.

To date, there is still a lack of sufficient studies to guide therapy for Bcc infections outside the respiratory tract.

The reported cases of *B. cepacia* bacteremia, osteomyelitis or abscesses, all showed susceptibilities to ceftazidime. The majority showed susceptibility to carbapenem and TMP-SMX as well. Fewer reports revealed susceptibility to quinolones, piperacillin-tazobactam and tigecycline [14, 15]. One reported patient had a recurrent *B. cepacia* infection causing osteomyelitis and a sternal abscess that was successfully treated with tigecycline [14]. Similar to the literature, the majority of collected Bcc cultures from all sites of our case series were susceptible to ceftazidime, followed by carbapenems and TMP-SMX. *B. cepacia* tends to be more resistant to quinolones and piperacillin-tazobactam. Most of our patients were successfully treated with single and combination regimens. The durations of therapy was variable depending on the host, the site of infection and the rapidity of clinical response. Those with osteomyelitis received a prolonged course of therapy as is the case in the management of osteomyelitis due to other pathogens. Since the antimicrobial stewardship program (ASP) efforts started at AUBMC in 2004, all the patients were followed closely by the Infectious Diseases team and the ASP team to guide the clinicians on proper duration of therapy. This was correlated with a positive outcome of the patients. Our current series can expand on the limited published reports on infections with Bcc outside the respiratory tract. We expect and hope that the next update of the IDSA guidelines on management of antimicrobial resistant Gram negative pathogens will include a section on Bcc infections to guide clinicians on the optimal therapy of such pathogens.

## Conclusion

*B. cepacia* infections whether bacteremia, SSTIs, abscesses or osteomyelitis were noted to be more common at our medical center as hospital-acquired infections particularly in Iraqi and Syrian patients. In addition, many of the patients had one or more immunosuppressive conditions. This raises the concern that patients in countries at war and with immunosuppressive conditions might be at increased risk for such infections. Empiric treatment for such pathogens might be needed in the right clinical scenario. There is a need to study the epidemiology of this pathogen in areas of wars and conflicts. Our results of antibiotic susceptibilities were consistent with the literature, showing that the majority of isolates are sensitive to ceftazidime, followed by carbapenems then TMP-SMX. Although *B. cepacia* is a resistant bacterium, almost all our patients were successfully treated under the guidance of an ASP. Future international guidelines are needed to guide clinicians for optimal therapy.

## Data Availability

The datasets during and/or analysed during the current study available from the corresponding author on reasonable request.

## Abbreviations

Bcc: *Burkholderia cepacia* Complex
AUBMC: American university of Beirut medical center
TMP-SMX: Trimethoprim sulfamethoxazole
CLSI: Clinical laboratory and standards institute
DD: Disc diffusion
EHR: Electronic health records
SOFA score: Sequential organ failure assessment score
SPSS: Statistical package for social sciences
ICU: Intensive care unit
SSTI: Skin and soft tissue infections
IDSA: The infectious diseases society of America
T5: Thoracic vertebrae number 5
ASP: Antimicrobial stewardship program
